# Online cognitive behavioral therapy enhanced for binge eating disorder: study protocol for a randomized controlled trial

**DOI:** 10.1186/s12888-020-02604-1

**Published:** 2020-04-29

**Authors:** Elske van den Berg, Bernou Melisse, Jitske Koenders, Margo de Jonge, Matthijs Blankers, Edwin de Beurs, Jack Dekker

**Affiliations:** 1 Novarum Center for Eating Disorders & Obesity, Jacob Obrechtstraat 92, 1071 KR Amsterdam, the Netherlands; 2 Research Department, Arkin Mental Health Institute, Klaprozenweg 111, 1033 NN Amsterdam, the Netherlands; 3 Section Clinical Psychology, Leiden University, Wassenaarseweg 52, 2333 AK Leiden, the Netherlands

**Keywords:** Randomized controlled trial, Binge eating disorder, Guided self-help, Cognitive behavioral therapy-enhanced, Waiting list

## Abstract

**Background:**

Binge eating disorder is characterized by recurrent episodes of binge eating accompanied by a sense of lack of control. Of the different treatments available, Cognitive Behavioral Therapy-Enhanced and guided self-help treatment are recommended. As online treatment offers several additional advantages, we have developed a CBT-Enhanced online guided self-help intervention. The aim of this study is to determine whether this intervention reduces eating disorder pathology and increases the amount of binge free days in adults classified with binge eating disorder or other specified feeding or eating disorder- binge eating disorder, compared to an untreated waiting list condition. The experimental condition is hypothesized to be superior to the waiting list condition.

**Methods:**

The efficacy of an online guided self-help intervention for binge eating disorder will be assessed by conducting a randomized controlled trial. The trial will target adult individuals classified with binge eating disorder or other specified feeding or eating disorder- binge eating disorder with a body mass index between 19.5 and 40, referred to an eating disorder treatment center. Dual arm allotment will be performed in a 1:1 ratio stratified for BMI above or below 30. Randomization will be blinded to the online intervention (*n* = 90), or to the control waiting list condition (*n* = 90). Assessors will be blinded and assessments will be administered at baseline, week 5, at end-of-treatment, and at 12 and 24 weeks follow-up. Primary outcome will be eating disorder pathology, operationalized as number of days on which binge eating occurred between the two conditions during the period of the intervention. Secondary outcome measures will be differences in other eating disorder pathology, clinical impairment and in quality of life, while therapeutic alliance, demographic characteristics and followed treatment module will serve as effect moderators. Several types of costs will be assessed.

**Discussion:**

This paper presents an online guided self-help Cognitive Behavioral Therapy- Enhanced study protocol for individuals classified with binge eating disorder or other specified feeding or eating disorder. Efficacy will be examined through a Randomized Controlled Trial.

**Trial registration:**

The study protocol is registered with the Netherlands Trial Registry NTR (NTR 7994) since 6 September 2019.

## Background

Eating disorders, one of the three most common disorders in adolescents [1], have a life time prevalence in the Netherlands of 1.74% [2]. These disorders, which have a significant impact on the psychological and physical well-being of affected individuals [3], are characterized by over-evaluation of shape and weight [4]. Binge eating disorder (BED) is specifically characterized by recurrent episodes of binge eating accompanied by a sense of lack of control [5]. The binges occur at least once a week and, if they are less frequent, the individual is classified with other specified feeding or eating disorder (OSFED) BED [5]. Of the population in the West, 2% suffers from binge eating disorder [6], and 1.5% from OSFED [7].

Of the range of treatments available for eating disorders, including Cognitive Behavioral Therapy (CBT), Dialectical Behavioral Therapy (DBT) and Interpersonal Therapy (IPT), Cognitive Behavioral Therapy-Enhanced (CBT-Enhanced) [8–10] is the preferred evidence based treatment for BED [9]. According to Fairburn, developer of CBT-Enhanced, [11, 12], CBT-Enhanced treatment leads to a recovery rate of 50–65% amongst the individuals with an eating disorder. For BED specifically, the recovery rate is 54% [12]. However, as, according to the stepped care principle, CBT-Enhanced could be too intensive for individuals suffering from BED or OSFED BED, international guidelines have recently recommended guided self-help treatment as first step for individuals suffering from BED [9]. Most online interventions are based on existing face to face treatment protocols [13]. Guided self-help has the advantages of reduced travel costs and travel time for the patient [14], less time of a specialist’s invested in a single treatment [15], and the removal of geographical distance as a barrier to seeking treatment [16]. In addition, an online guided self-help version of CBT-Enhanced (GSH CBT-E) follows all recommended evidence-based guidelines for individuals suffering from BED [9].

Studies have shown that several online self-help treatments, including the use of apps on different devices such as smartphones or computers, are effective when combined with therapeutic support [17]. This is also the case for BED [17, 18], and a recent meta-analysis showed that 46% of the individuals classified with BED report not to suffer from binge eating episodes after self-help treatment [19]. There are, however, differences in the efficacy of self-help treatment [18], with guided self-help (GSH) being found to be more effective than non-guided self-help [20], and a stronger therapeutic alliance improving treatment efficacy [21, 22]. The literature further suggests therapeutic alliance to be stronger when contact is synchronous as by phone than when asynchronous as by email [21].

Responding to the absence of CBT-Enhanced based guided self- help (GSH) in Dutch, Novarum, Center for Eating Disorders in the Netherlands, has developed a CBT-Enhanced based GSH (GSH CBT-E), based on the self-help section (Part two) of *Overcoming Binge Eating, The Proven Program to Learn Why You Binge and How You Can Stop* [23]. A study of GSH for BED by Ter Huurne et al., based on general Cognitive Behavioral Therapy (CBT) and Motivational Interviewing (MI), in the Netherlands have shown that, compared to a waiting list condition, an online GSH version of Cognitive Behavioral Therapy (GSH CBT), is particularly effective in reducing eating disorder pathology. At the same time, reduction in binge eating episodes, the main symptom in individuals suffering from BED, has not been reported [24]. Although one GSH CBT study in Germany did show a reduction of binge eating episodes of 58% [25], the treatment protocols used in both studies, in the Netherlands as well as in Germany, were not based on CBT-Enhanced, and a GSH version of CBT-Enhanced is expected to be more effective than GSH CBT [21, 26].

To broaden the treatment method to CBT-Enhanced, in this study, contact with the participants in this study will be synchronous, using direct phone contact, unlike in Ter Huurne et al. [24], where patient contact was asynchronous, through email [21, 22, 24]. Again extending Ter Huurne et al. [24], which was conducted with only female participants [24], this study will include both male as well as female participants. Thus, even though gender is not expected to influence treatment efficacy [27], the results will be relevant to both genders. Finally, where Ter Huurne et al. [24] used self-report questionnaires to classify study participants and assess eating disorder pathology, including pre- and post-treatment binge eating behavior, this study will use the Eating Disorder Examination (EDE) interview [28] as well as self-report questionnaires [29].

The primary aim of this study is to examine the efficacy of online GSH CBT-E with regard to eating disorder pathology, operationalized as difference in the number of binge-free days among men and women classified with BED and/ or OSFED BED after treatment compared to a waiting list control group. The secondary aim is to examine the efficacy with regard to other eating disorder pathology, clinical impairment and quality of life between GSH CBT-E and a waiting list control group after treatment and during the follow-up period. Tertiary aim is to calculate direct treatment costs for the GSH CBT-E intervention for means of transparency, direct comparison with the waiting list condition, and indirect comparisons with other interventions. Efficacy will be examined through a parallel group randomized controlled trial.

It is hypothesized that GSH CBT-E is superior to the waiting list condition with regard to a decrease in eating disorder pathology and clinical impairment. GSH CBT-E is also expected to be superior to the waiting list with regard to increase in the number of binge free days and quality of life.

## Methods

### Trial design

**Fig. 1:**
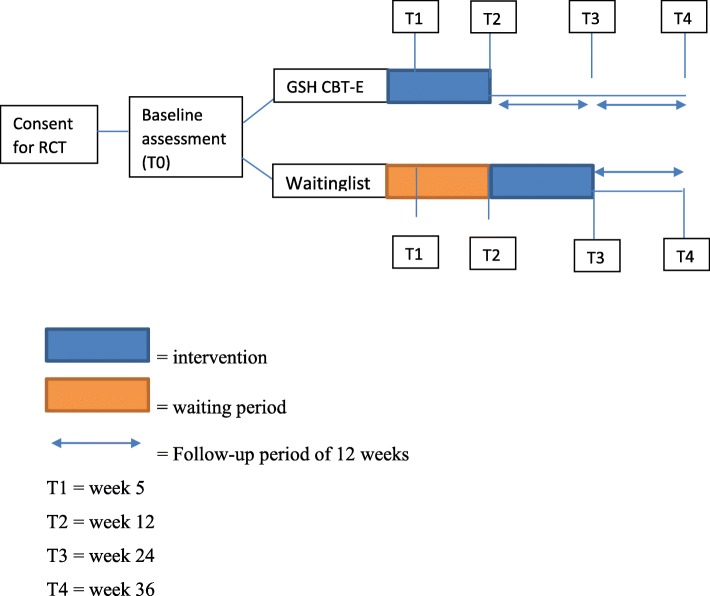
Flow chart of planned intervention and assessment

A single center randomized controlled trial (RCT) will be conducted at Novarum, center for eating disorders in Amsterdam, the Netherlands. Participants in the experimental condition (GSH CBT-E) will be compared to the waiting list control condition. To our knowledge this is the first study examining efficacy of GSH CBT-E, since only efficacy results of GSH CBT have been reported [30–32]. Therefore, outcome of GSH CBT-E will first be compared to waiting list control condition. Figure 1 presents a flowchart of this study.

Participants are referred for treatment by general practitioners, secondary care health professionals or general hospitals. All potential participants are over 18 years of age and are being treated voluntarily. All potential participants classified with BED or OSFED BED, assessed by a clinical psychologist or psychiatrist, are invited to participate in the study. If a potential participant meets all inclusion and none of the exclusion criteria, agrees to the terms and conditions of the study and has provided informed consent, a baseline assessment (T0) will be scheduled. After the baseline assessment is completed, participants will be randomized to either the experimental condition (GSH CBT-E) or the waiting list control condition. Randomization will be blinded and stratified for body mass index (BMI) above or below 30. BMI will be calculated as weight in kg/ height in m^2^. Participants assigned to the experimental condition will start their treatment directly after they read the mandatory literature. The control group will start treatment after a waiting list period of 12 weeks, the same duration as the intervention. After baseline (T0), assessments will take place four times during and after treatment: at week 5, which is the evaluation-point of treatment for GSH CBT-E (T1); at the end of treatment for GSH CBT-E/ start of treatment for waiting list control group (week 12, T2); at 12 weeks after treatment for GSH CBT-E/ End of treatment for the waiting list condition (week 24, T3); and at 24 weeks after treatment for GSH CBT-E/, 12 weeks after treatment for waiting list control group (week 36, T4). The initial screening, before consent to study participation will take place at Novarum, but the intervention and assessments will take place at any location where participants have internet connection and are able to focus on their sessions. Since a large proportion of the Dutch population (96.7%) had internet access in 2018 [33], the participants’ personal location can be anywhere in the Netherlands. Obtained close- out period will be 1.5 years.

### Participants and recruitment

#### Inclusion

In order to be eligible to participate in this study, a participant must meet the following criteria:

1. DSM- 5 BED or OSFED BED classification;
2. age at least 18;
3. BMI between 19.5 and 40;
4. moderate proficiency in Dutch and ability to read part one of the Dutch translation of *Overcoming Binge Eating, The Proven Program to Learn Why You Binge and How You Can Stop*, by Christopher Fairburn [34], which involves psychoeducation;
5. willingness to provide contact details, including phone number, internet access, possession of a computer or tablet, and willingness to use it for treatment and research purposes;
6. willingness to sign the informed consent form.

#### Exclusion

Primary exclusion criteria are:

1. acute psychosis, assessed via Structural Clinical Interview by the DSM 5 (SCID-5) [35];
2. acute depression, assessed via SCID-5;
3. suicidal ideation, assessed via SCID-5 or
4. self-induced vomiting as compensatory behavior, as reported at initial session or EDE-interview at baseline.
5. having received eating disorder treatment during the past 6 months,
6. being pregnant
7. reported use of medication with the potential to influence eating behavior such as, Lithium, Mitrazepine and anti-psychotic stimulants [36, 37].

#### Recruitment

Recruitment will take place between September 2019 and December 2020. Potential participants will be referred to Novarum, center for eating disorders by other health care specialists. After being referred for specialized eating disorder treatment, potential participants have an initial screening consisting of an interview about eating disorders symptoms, other general psychiatric symptoms, life events and demographics. Weight and height will be measured in order to calculate their BMI. All eligible potential participants receive written study information during an advisory session, during which they receive an informed consent description, explaining the research goals and information about participation in the study. When willing to participate they sign the informed consent form and their baseline assessment will be planned. As an incentive for participating, participants will receive €10,- in gifts cards after completion of T2 (12 weeks), after completion of T3 (24 weeks) and €20,- after measurement at T4 (36 weeks). Participants who complete all follow-up questionnaires will thus receive a total amount of €40 in gifts cards as compensation for participating in the study.

### Intervention and procedure

All participants will receive GSH CBT-E: the experimental group will receive treatment immediately after baseline assessment, and the control group after a 12 weeks waiting period. GSH CBT-E is an online guided self-help version of CBT-Enhanced [8], based on *Overcoming Binge Eating, The Proven Program to Learn Why You Binge and How You Can Stop* [23], whose second part, a self-help guide, has been transformed into an online intervention.

#### Development of the intervention

The GSH CBT-E protocol was converted into an online program by CBT-Enhanced trained specialists at Novarum, center for eating disorders. A software team implemented the protocol on a website. During the pilot phase of the initial version, through an interactive process involving participants and therapists feedback, the website was further developed in terms of user friendliness, ease of navigation, lay-out and reduction in eating disorder pathology, measured by the Eating Disorder Examination-Questionnaire (EDE-Q) [29]. A risk inventory was performed [38] and GSH-CBT-E appeared to be a medical device involving minimal risks, therefore it was not necessary to gain permission with the Dutch Health Care Inspectorate [39].

#### Procedure

##### Therapists

Therapists activate an account and participants log into their digital environment through my.karify.com. Both categories have to register with a personal username and password. Both categories are able to access the intervention at any time. Once the patient completes an assignment, the therapist receives a notification by email and can access the assignments. All therapists are trained CBT-E therapists, successfully completed a web based CBT-Enhanced training provided by Centre for Research on Eating Disorders at Oxford (CREDO) and worked through the detailed CBT- Enhanced guide [8]. The therapists have different disciplinary backgrounds and have completed a post doc degree (mental health care psychologist), a masters degree (psychologist), or a bachelors degree (dietician and social worker). A manual explaining all intervention modules in detail is available to all GSH CBT-E therapists. After completing their CBT-Enhanced training by CREDO, all therapists received 2 days of training, including background information, program navigation, working with scripts, treatment content and communication skills. All therapists will receive supervision once a week, including general guidelines, discussion of adverse events and participants’ treatment progress. Therapists will not be blinded.

##### Intervention

GSH-CBT-E is based on Cognitive Behavioral Therapy- Enhanced, focused version, offered in a guided self-help format consisting of CBT-Enhanced key interventions, inevitably however less complex and with a shorter duration as in CBT-Enhanced focused. Before participants are eligible to start treatment, they have to read Part one of *Overcoming Binge Eating, The Proven Program to Learn Why You Binge and How You Can Stop* [23], or the Dutch translation *Overwin je eetbuien, waarom je te veel eet en hoe je daar mee kunt stoppen* [34]*.* This contains psycho-education about eating disorders and will be referred to during treatment. Participants will be asked if they have read part one before start of treatment. If the patient has not, they will be asked to read it during the first week(s) of treatment. In addition, before starting treatment, baseline assessment will take place. The EDE will be conducted by phone and participants will be sent a link to the self-report questionnaires, which they can complete at home on a computer. After baseline assessment and reading part one of *Overcoming Binge Eating The Proven Program to Learn Why You Binge and How You Can Stop* [23], the patient will be ready to start GSH CBT-E. Just like regular CBT-Enhanced, GSH CBT-E comprises three main stages; the first stage focuses on establishing regular eating and alternatives for binge-eating, using real-time self-monitoring as central intervention, and events, moods and eating. After joint review of progress & designing rest of treatment in the second stage, the third stages focuses on dietary restraint or shape concern and finally ending well with a firm focus on minimizing the risk of relapse in the long term. GSH CBT-E is a 12 week program. Once a week there will be a therapy session. All sessions will be conducted by phone. Participants have to start reading information online, to monitor their eating behavior, and to schedule, once a week, weighing, and, twice a week, self-evaluation sessions. The program is interactive, participants have to upload assignments and the therapist can monitor them. In addition, patient and therapist do interact weekly. Therefore, it differs from merely reading an e-book. A few days after start of treatment, participants will have a 20 min phone session with their therapist. To ensure consistency between therapists, these phone sessions are pre-scripted. During the first 4 weeks, participants will have to monitor their eating behavior, including their thoughts and feelings and establish a regular eating pattern. They will also introduce alternative activities for binge eating and work on problem-solving skills. During week 5, they will fill out assessment questionnaires, sent to them by a link, and their progress will be assessed, by both themselves and their therapist. This session will enable them to decide whether to add a module on body evaluation or dietary restraint during week 6–11 in addition to monitoring, regular eating, alternatives for binge eating and problem solving (Table 1). From week six onwards the program modifies to the user, based on the maintaining factors of their binge eating disorder. Before the 12th session participants will have to fill out the questionnaires. Results will be discussed during session 12, when they will also discuss what to do to prevent set-backs. The post treatment EDE will be conducted by phone. GSH CBT-E treatment will not be altered or interfered with during the study. As GSH CBT-E is a 100% guided self-help treatment without face to face sessions. After conclusion of the treatment, participants can still access the treatment module. To ensure data protection GSH- CBT-E is only available through a HTTPS protocol and secured by a 256-bit encrypted SSL- certificate. The online environment (Karify) is ISO 27001 and NEN 7510 certified.

**Table 1:** Overview of timing of interventions in GSH CBT-E

Step	Focus	Period (12 weeks total)
Step 1	Starting well	1 week
Step 2	Establish a regular eating pattern	1 week
Step 3	Alternatives for binge eating	1 week
Step 4	Problem solving	1 week
Step 5	Evaluation	1 week
Step 6	Module: dietary restraint	Distributed over 6 weeks
Step 6	Module: shape concern	Distributed over 6 weeks
Step 8	Ending well	1 week

However, when another disorder than BED becomes the participants’ primary complaint and interferes with GSH CBT-E this will be considered as an adverse event. When the participant meets the criteria of Bulimia Nervosa instead of Binge Eating Disorder, as reported at the initial session or EDE-interview at baseline [40] the participant will no longer partake in the protocol and will be offered face to face treatment. In addition, patients will report on the frequency of their eating disorder behaviors on a weekly basis, monitored by their therapist. Although sessions are conducted by phone, all therapists will pay close attention to adverse events and patients will receive face-to-face treatment when dealing with adverse events. If presence of another psychiatric disorder interferes with GSH CBT-E, the participant will first consult a psychiatrist and receive face to face treatment or will be offered treatment in or outside our institutions.

##### Experimental treatment group

Participants assigned to the experimental condition will start their GSH CBT-E intervention directly after baseline assessment.

##### Waiting list control group

Participants assigned to the non- experimental (waiting list) condition will start their GSH CBT-E treatment 12 weeks after baseline. This control condition will be a minimal intervention. Participants will be called every 6 weeks for a short conversation (10 min at most), which will include checking on the eating disorder symptoms and other important areas of the participant’s life. This is necessary as, under Dutch law, monitoring participants during their waiting list period is mandatory at least every 6 weeks.

### Outcome measures and assessment

#### Outcome measures

##### Primary outcome measure

The primary outcome measure will be difference in eating disorder pathology, operationalized as the amount of days binge eating occurred, post treatment versus the waiting list control condition. Due to the absence of purging behavior, participants experience difficulty to recall the number of binge eating episodes. Therefore, participants suffering from BED were asked to report the number of days binge eating occurred rather than number of episodes [26, 41]. This is in line with the primary outcome measure chosen in another study [41]. Amount of binge free days will be measured by the EDE [28] and EDE-Q [29]. If a discrepancy arises between binge free days on the EDE and EDE-Q, the EDE will be chosen over the EDE-Q, because it is the golden standard for assessing eating disorder pathology [42].

##### Secondary outcome measure

As secondary outcomes, differences in other eating disorder pathology defined as restraint, eating concern, weight concern and shape concern [29, 40], quality of life and clinical impairment will be assessed. Eating disorder pathology will be measured by the EDE [28] and EDE-Q [29]. Quality of life will be measured by the five-level variant of the five-dimensional EuroQol instrument (EQ-5D- 5 L) [43]. Clinical impairment will be assessed through the Clinical Impairment Assessment (CIA) developed by Bohn et al. [44].

##### Costs

The economic evaluation will be performed in line with the ISPOR guidelines [45] and the Consolidated Health Economic Evaluation Reporting Standards (CHEERS) statement [46]. Several types of costs will be assessed: utilization of health care, costs stemming from productivity losses due to absenteeism or reduced efficiency while at work (presenteeism) and GSH CBT-E costs. Utilization of health care, other than for GSH CBT-E and utilization of health care within Arkin will be assessed by the questionnaire for Costs associated with Psychiatric Illness (TiC-P) [47]. Health care use will be multiplied with standard cost prices [48]. In addition, the participants electronic file will be searched to make additional calculations on costs other than GSH CBT-E within Arkin. Regarding productivity losses, we will use the Dutch guideline for economic evaluation [49], and rely on the standard cost prices reported therein. Productivity losses will be based on the gender- and age-specific friction costs. In addition, costs of GSH CBT-E (expressed in euros) will be established for each participant by multiplying standard Dutch cost prices [49], with the number of patient contacts. Since the time horizon of this study, mean time frame between start and end of treatment, is under a year, no discounting for future costs / effects will be applied.

##### Other study outcome measures

The variables that will be investigated as moderators of treatment effect are: therapeutic alliance, demographic variables, and the online module followed by the participant (dietary restraint or shape concern). Therapeutic alliance, hypothesized to be a moderator, will be measured through the Working Alliance Inventory (WAI) [50]. Gender is not expected to influence treatment outcome [27].

#### Assessment

**Fig. 2:**
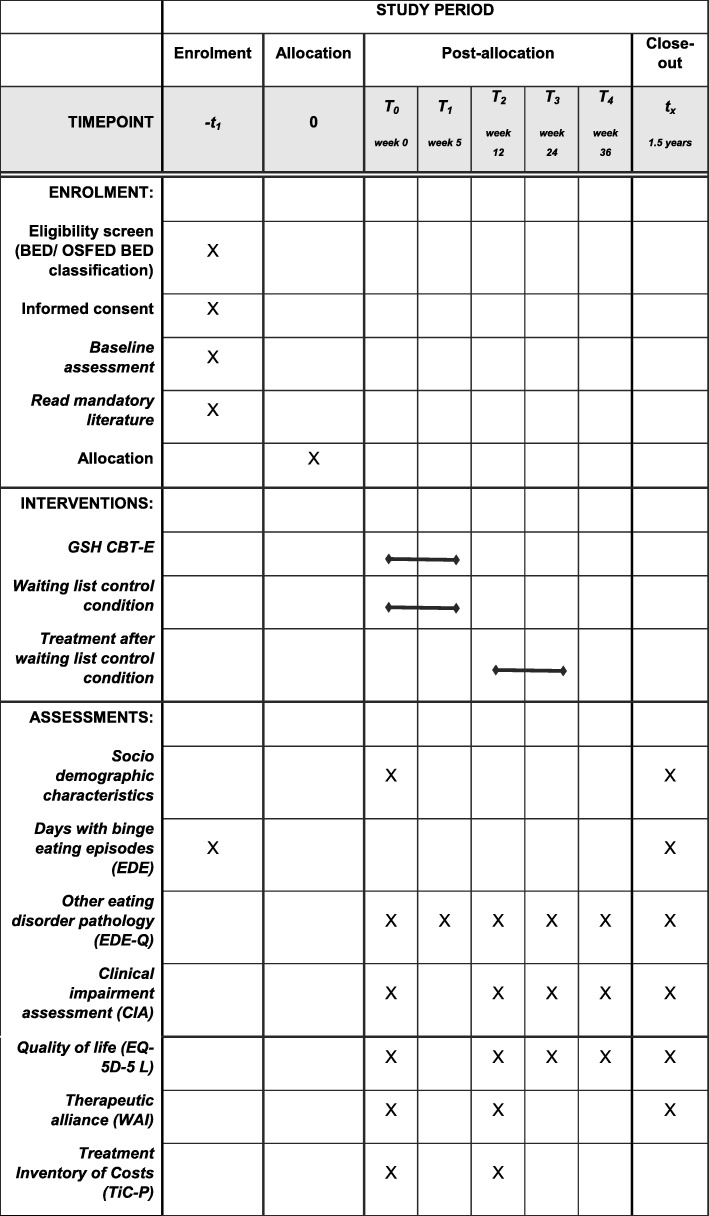
Schedule for timing of the intervention and different assessments

All assessments will be conducted by phone (EDE) or via a link provided by e-mail (EDE-Q, CIA, EQ-5D-5 L, WAI). The EDE will be conducted at start (week 0) and post treatment (week 12). These assessments will be conducted by research assistants who will be blinded. If blinding is broken, the assessment will still be completed, and the results compared with results of the other participants in the same condition. If the results of the participant with broken blinding are outliers, they will not be taken into account in the analyses. All other assessments will be conducted at start, during week 5, week 12 and at week 24 and 36 (see also Figs. 1 and 2). Since treatment effect is expected to continue after end of treatment [11, 12], follow up measures will be conducted. If a participant does not complete the online assessments they will be called by a research assistant in order to motivate them. All assessments will be processed in Castor EDC (https://www.castoredc.com/) which is ISO 27001/27002/9001 and NEN 7510 certified. In addition, staff conducting the assessments do not offer GSH-CBT-E themselves.

#### Assessment of primary and secondary outcomes: number of binge free days and other eating disorder pathology

##### EDE

The Eating Disorder Examination (EDE) [28] is a semi- structured interview assessing eating disorder pathology during the last 28 days, including binge eating behavior. The EDE, considered to be the golden standard for eating disorder assessment, has good discriminative and concurrent validity [51], internal consistency [28], and test-retest reliability [52]. Eating disorder symptoms are measured on a 7 point Likert scale [40].

##### EDE-Q

The Dutch version of the Eating Disorders Examination-Questionnaire 6.0 (EDE-Q) [29, 53] will be used to asses eating disorder pathology. The EDE-Q, consists of four subscales: restraint, eating concerns, weight concerns and shape concerns [29]. The Dutch version of the EDE-Q, has good psychometric properties [53]. The EDE-Q is a self-report questionnaire of 36 items, measuring bingeing behavior during the last 4 weeks. Eating pathology will be measured on a 7 point-Likert scale [29].

#### Assessment of secondary outcomes: quality of life and clinical impairment

##### EQ-5D-5L

Quality of life will be measured by the five-level variant of the five-dimensional EuroQol instrument (EQ-5D-5 L) [43]. A Dutch version of the EQ-5D-5 L has been made available [54]. Psychometric properties for the Dutch version are known to be reliable for a set of different countries, including the Netherlands [55].

##### CIA

Due to eating and compensatory behavior as well as concerns regarding shape, weight and eating patterns, eating disorders occur with several psychosocial impairments. In this study, clinical impairment will be assessed through the Clinical Impairment Assessment (CIA) developed by Bohn et al. [44], which differentiates between eating disorder psychopathology and the impairment secondary to ED psychopathology. The CIA assesses clinical impairment across specific areas of life: personal, social and cognitive. The CIA is a 16 item self-report questionnaire, with items rated on a 4 point Likert scale. The Cronbach’s Alpha is .97 and convergence validity .68 [44]. This study will employ the Dutch translation, which has been shown to have good psychometric properties [56].

#### Costs

##### TiC-P

Health care costs and productivity gains/losses will be measured using the TiC-P [57]. The TiC-P is a self-report questionnaire measuring healthcare consumption and production losses as a consequence of psychiatric disorders. The first part of the TiC-P includes 14 structured no/yes questions on the amount of health care utilization. The second part measures productivity gains/losses by measuring absence from work and reduced efficiency of paid and unpaid work [47, 48, 57].

#### Other measures regarding the intervention

##### Demographics

Demographic characteristics, including age, gender, marital status, domestic situation, level of education and occupational/ student status, will be asked of the participants. These socio-demographic characteristics, which will serve as a moderator, will be assessed by phone during the baseline assessment.

##### Therapeutic Alliance (WAI)

Therapeutic alliance will be measured through the Working Alliance Inventory (WAI), a 36- item self-report questionnaire addressing working alliance between patient and therapist. The items are scored on a 7 point Likert scale, resulting in three dimensions: Bonds, Tasks and Goals [50]. The WAI has high internal reliability [58] and Cronbach’s Alpha is 0.95 [59]. In this study, only the patient’s perspective will be assessed.

### Sample size and power calculation

Effects of GSH CBT-E are expected to be comparable to other self-help treatments targeting eating behavior, i.e. a 46% decrease in binge eating episodes with an effect size of Cohen’s *d* = 0.47 between the experimental and control condition [19]. Since self-help treatment for BED has an overall drop-out of 24% [19], 25% more participants are included to obtain sufficient power. Sample size calculation has been conducted using R package ‘pwr’. The sample size without correction is *N* = 144 (*n* = 72 per arm). The sample size corrected for drop-out will be *N* = 180 (*n* = 90 per arm), with a power of 80%, to find an effect size of *d* = 0.47, α = 0.05 (2-sided).

### Randomization

Randomization will take place after baseline assessment. Dual arm allocation will be performed in a 1:1 ratio, stratified for BMI score. Strata will be defined based on BMI: 19.5 ≥ BMI < 30 or 30 ≥ BMI < 40 by randomly selected block sizes of four, six and eight. Randomization will be performed digitally using Castor EDC (https://www.castoredc.com/) by an independent Arkin data processor not involved in this study. Participants will be informed about their allocation by email by the second author. All other authors will be blinded.

### Statistical analysis

The number of binge free days, other eating disorder pathology, quality of life, clinical impairment and therapeutic alliance will be reported as means (standard deviations); and effect sizes between the two conditions will be reported as Cohen’s *d*. Direct costs of the eating disorder treatment will be reported in euros. Cost-effectiveness analysis with number of binge- free days as effect measure and a cost- utility analysis using QALYs will be performed. The QALYs will be derived from the Dutch version of EQ-5D-5 L [43]. Generalized Linear Mixed Models (GLMM) will be used for the analysis of intervention outcomes. The variable BMI 19.5 ≥ BMI < 30 and 30 ≥ BMI < 40 will be included in the GLMM models as covariate. Analyses will be conducted on the entire randomized sample (i.e. intention to treat), and on the per protocol/ treatment completers sample. The primary endpoint for the study is 12 weeks post-randomization. As there are no multiple primary endpoints, there will be no Bonferroni correction or other correction for the significance level applied. Missing data will be handled using multiple imputation. All analyses will be carried out using SPSS version 22+ and R version 3.0+ by an independent Arkin data processor. Results are planned to be published in international peer reviewed journals.

### Ethical approval

Study approval was given in August 2019 by the Medical Research Ethics Committees United (*MEC*-*U*) (reference number NL 6958.100.19) in Nieuwegein, the Netherlands.

## Discussion

This paper presents a study protocol of an RCT on the efficacy of online GSH CBT-E treatment for individuals suffering from BED and/or OSFED BED. GSH CBT-E is the first treatment in the Netherlands to follow all NICE [9] guidelines for BED: according to these guidelines is CBT-Enhanced the preferred treatment and that treatment should be offered through the stepped care principle, and recommending guided self-help treatment. The primary goal of GSH CBT-E is to increase the amount of binge free days; secondary goals are to decrease eating disorder pathology and clinical impairment related to the eating disorder and to improve quality of life. Short term efficacy will be assessed directly post treatment, long-term effects will be assessed 12 and 24 weeks post treatment. Direct treatment costs of the intervention will also be calculated.

GSH CBT-E has several strengths. A guided self-help variation of CBT-Enhanced, it meets all recent guidelines for individuals suffering from BED. In addition, since participants can participate in their personal environment, GSH- CBT-E enables individuals to overcome barriers they may experience with face to face treatments, such as stigma, travel time and costs and availability [14–16]. GSH CBT-E will also reduce specialist’s time invested in a single treatment [15]. Based on the number of referrals, we expect to include 12 patients a month, which should be sufficient to result in a final sample size of 180 participants, allowing for a drop-out rate of 25% [19].

This study may also face some challenges. Participants and therapists might not feel comfortable working in an environment that is solely online and phone based due to a lack of face to face contact, or because they might face challenges navigating within the program. Therapists and participants might also experience issues with wireless connection, reception and malware. Therapists may also find it challenging to adapt to the role of guiding self-help therapy rather than providing face to face therapy. Although after the initial session, there is no face to face contact between assessors, therapists and participants, attention will be paid to adverse events, harms or differences in disease state.

If proven effective, evidence-based online GSH CBT-E treatment meeting all NICE [9] guidelines can be offered in the Netherlands. This will potentially extend treatment availability for individuals classified with BED and/or OSFED BED, reduce waiting lists and decrease costs of offering and receiving treatment. If GSH CBT-E is indeed effective in treatment of BED and/or OSFED BED, it will be the first evidence based guided self-help treatment based on CBT-E in the Netherlands. Results from this study will be provided according to the SPIRIT guidelines.

## Data Availability

Not applicable.

## Abbreviations

BED: Binge eating disorder
CBT: Cognitive Behavioral Therapy
CBT-Enhanced: Cognitive Behavioral Therapy- Enhanced
CIA: Clinical Impairment Assessment
DBT: Dialectical Behavioral Therapy
EDE: Eating Disorder Examination
EDE-Q: Eating Disorder Examination-Questionnaire
EQ-5D-5 L: EuroQol five-dimensional five-level
GSH CBT-E: Guided Self-Help Cognitive Behavioral Therapy- Enhanced
IPT: Interpersonal Therapy
MI: Motivational Interviewing
NICE: National Institute for Health and Care Excellence
OSFED: Other specified feeding or eating disorder
RCT: Randomized Controlled Trial
SCID-5: Structured Clinical Interview for DSM-5
SPIRIT: Standard Protocol Items: Recommendations for Protocol Trials
WAI: Working Alliance Inventory
